# Change management in higher education: A sequential mixed methods study exploring employees’ perception

**DOI:** 10.1371/journal.pone.0289005

**Published:** 2023-07-21

**Authors:** Rima Ezzeddine, Farah Otaki, Sohaib Darwish, Reem AlGurg

**Affiliations:** 1 Strategy and Institutional Excellence, Mohammed Bin Rashid University of Medicine and Health Sciences, Dubai, United Arab Emirates; 2 College of Medicine, Mohammed Bin Rashid University of Medicine and Health Sciences, Dubai, United Arab Emirates; Alexandria University Faculty of Nursing, EGYPT

## Abstract

**Background:**

Higher education institutions need to put change management as a pivotal part of their strategy. The challenge is to effectively contextualize existing change management models to the respective work environment. Failing to properly adapt existing models to match the intricacies of the environment could lead to plenty of setbacks. For such a contextualization to take place, gauging employees’ engagement and satisfaction becomes of paramount importance. As such, the overall purpose of the current study is to explore the perception of employees of a medical and health sciences university in Middle East and North Africa (MENA) region, in relation to change management and agility, and to showcase how the captured perspectives can be systemically interpreted to inform decision-making in the context of the study.

**Method:**

This research study relied on a sequential mixed methods design, which started with an exploration of the perception of Mohammed Bin Rashid University of Medicine and Health Sciences (MBRU) leaders. Qualitative data was collected through a focus group session and was inductively analysed (based on constructivist epistemology). The output of the qualitative analysis contributed to the development of the quantitative data collection tool. The quantitative data was analysed by SPSS-version-27.

**Findings:**

The qualitative analysis generated three key themes: Trigger, Execution, and Results, along with a thorough outline of lessons learned and opportunities for improvement. The Cronbach’s Alpha reliability score was 92.8%. The percentage of the total average of agreement was 72.3%, and it appeared that 83.2% of the variance can be explained by the instrument (p<0.001).

**Conclusion:**

The current study generated a novel conceptual framework that can be leveraged by educational leadership and administration to reinforce their decisions and optimize their agility in terms of managing change. The study also introduces a data collection tool which captures the perception of higher education stakeholders regarding the way their respective institutions handle change. This tool proved to be reliable and valid in the context of the study.

## Introduction

As suggested by the Greek philosopher, Heraclitus: “change is the only constant”. As such, it is of utmost importance in life to be open and adaptable to change [1]. This becomes particularly relevant in the business world, where effectively managing change within organizations can lead to many positive aspects, such as retaining a competitive edge and remaining relevant in one’s business area. Change also encourages innovation, develops skills and employees’ morale, and leads to better outcomes [2]. In fact, today’s world is commonly described using the acronym: VUCA-Volatility, Uncertainty, Complexity, and Ambiguity. This acronym was initially coined in the military context [3].

There are many reasons to change. Change usually occurs in response to internal and/ or external stimuli. An internal trigger could be a change in the organizational structure or strategy (e.g., merging and/ or acquisition) [4]. As for external triggers, these might include technology advancements [5], changes in governmental regulations [6], or crises, such as COVID-19 [7].

Our world was already experiencing an extraordinary rate of change at the onset of the pandemic, and since then, the speed at which change has been occurring became even higher. This can be attributed to many factors including but not limited to those related to technology advancement and changes in human ideologies [8]. How we think about vital concepts such as our identities, globalization, and sustainability has drastically changed, as well. While change is central to humanity, as previously mentioned, today’s ever-changing world requires a new, more sophisticated paradigm of change management, in general, and of leadership, in specific. As such, organizations need to put change management as a pivotal part of their strategy.

This is of great relevance to the higher education sector, which is characterized by several unique drivers of change including its connection to the Global, knowledge economy and the growing public accountability [9, 10]. Higher education is under increasing scrutiny to demonstrate greater accountability and transparency. The increasing diversity of students and corporatization of learning environments are other major drivers of change [11]. There is also the rising competition and for-profit education, along with the everchanging understanding of learning and teaching, the internationalization of campuses, and the rise of innovative technologies [12], where universities are enabled to run more efficiently and to educate students more effectively [13, 14]. All this is compounded with the drastic pressure that was imposed due to the pandemic and the changes that have been taking place since then. As such, higher education institutions are caught in a critically demanding and increasing unknown present and future characterized by VUCA-Volatility, Uncertainty, Complexity, and Ambiguity [3]. There are different approaches, models, and formulas that organizations, in higher education or otherwise, can adapt to effectively manage change. These include ones that perceive leading change as a process such as that of John Kotter’s [15, 16]. Among those models that discuss the process integral to change are those that reflect upon the dismantling that is necessary to happen prior to the change such as that of William Bridges, which describes the importance of letting go as a prerequisite to the new beginning, and that introduced by Kurt Lewin, which uniquely highlights the importance of unlearning previously adapted modi operandi [15, 17]. There are models that discuss enablers to change such as those of Beckhard and Harris, and ADKAR introduced by Hiatt [18]. Along the same lines, there is that introduced by Knoster [19] which suggests that for change to be effectively managed 6 elements need to come together, namely: Vision, Consensus, Skills, Incentives, Resources, and Action Plan.

It is evident that the literature is full of models, but the challenge is to effectively contextualize these models to the respective working environment. The model, in of itself, can be great but then if it is not properly adapted to match the intricacies of the environment, this could lead to plenty of setbacks and become counterproductive. The more complex an organization is, the more important it is to tactically adapt any existing change management theory, concept, and/ or model. For such a contextualization to take place, gauging employees’ engagement and satisfaction becomes of paramount importance. As such, the overall purpose of this research project is to explore the perception of employees of a medical and health sciences university in the MENA region in relation to change management and agility, and to showcase how the captured perspectives are systemically interpreted to inform decision-making in the context of the study. The research questions are:

- How do the employees perceive change and its management, in general, and within their higher education institution, in specific?
- How efficacious was the management of change from the perception of the participating employees?
- What are the employees’ preferences when it comes to management during times of change?

## Methods

### Context of the study

The current study took place in the Mohammed Bin Rashid University of Medicine and Health Sciences (MBRU) [20–23]. MBRU was established in 2016 with the aspiration of becoming a global hub for innovative and integrated healthcare education and research at the service of humanity. MBRU’s mission is to advance health in the UAE and the region, through an innovative and integrated academic health system, that is nationally responsive and globally connected, serving individuals and communities [24]. MBRU is an inclusive educational institution, comprising of a diverse faculty and student body featuring more than 32 nationalities [25, 26]. MBRU has 3 colleges, which offer several nationally accredited and internationally recognized educational programs. It has firm affiliations with a myriad of local and international stakeholders [27–29].

This context has been undergoing drastic changes to first quickly grow from a start-up medical school to gain national prominence and global impact, to rapidly transition to distance learning due to the unprecedented COVID-19, and more recently to become one of the key players of the novel Dubai Academic Health Corporation: an academic health systems, which is characterized by tripartite missions of medical and health professions’ education, research and knowledge generation, and clinical practice.

### Research design

**Fig 1:**
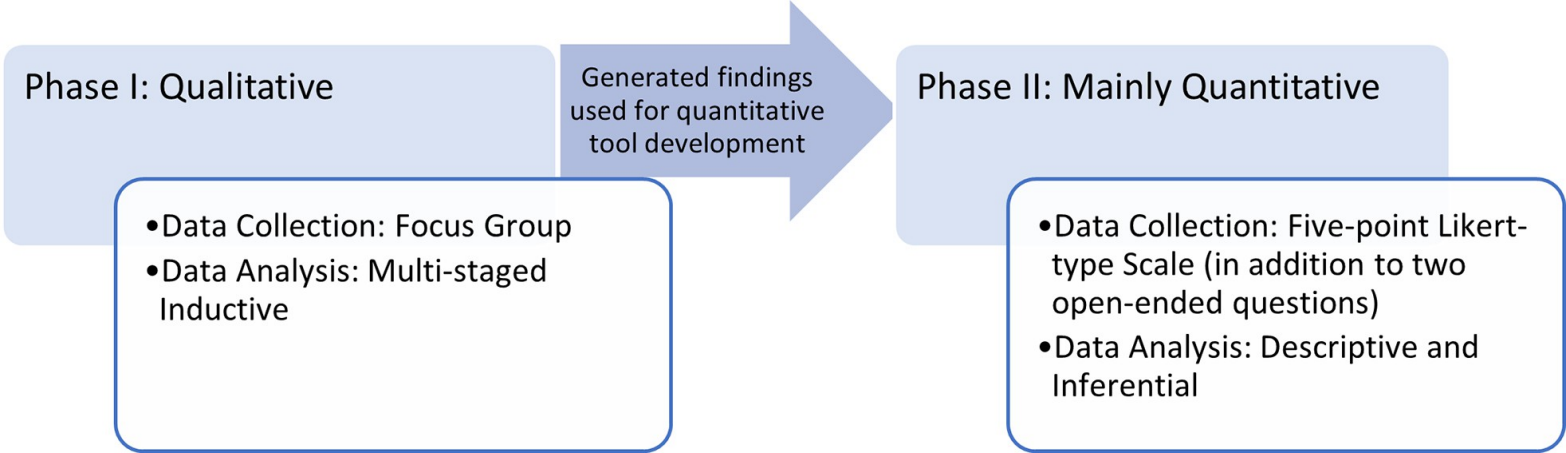
Outline of research design.

This study relied on an exploratory sequential mixed methods research design [30–32], composed of two discrete phases (Fig 1). The first phase was qualitative and meant to explore stakeholders’ perception of the subject matter. The second phase was quantitative, relying on a data collection tool that was developed based on the findings generated from the preceding phase. As such, the second phase was meant to investigate outstanding findings of the first phase, along with capturing complementary information. An electronic consent was obtained from each participant, where they had complete autonomy to choose whether, or not, to participate. This study was approved by the Institutional Review Board of MBRU (MBRU-IRB-2021-054).

### Data collection and analysis

#### Qualitative

The research study started with an exploration of the perception of the MBRU Key Opinion Leaders (KOLs) regarding agility and change management. Qualitative data was collected through a focus group session of randomly selected KOLs (i.e., directors of organizational units/ higher management); 10 were invited to participate. A tailor-made focus group protocol was developed for the purpose of this data collection initiative (S1 Appendix). The data collection tool underwent two validation phases. First, the current study researchers reached out to three experts in the field of change management to solicit their informed support in conducting content validity. Second, the questions of the data collection tool were discussed with six members of the University’s community, who are not among the University’s higher management team (two middle managers, two faculty members, and two students) to assess the clarity, readability, and comprehensibility of the questions, and the flow by which they are presented (i.e., face validity).The focus group protocol was composed of four segments. The first one explores the stakeholders’ general understanding of key concepts related to effectively managing change in institutions. The second segment of the protocol is more focused on change management in the context of MBRU. As for the third segment, it was evaluative in nature, where the stakeholders were asked to collectively identify the strengths, weaknesses, opportunities, and threats of their first-hand experience with change management at MBRU (throughout their tenures, respectively). Finally, the last segment of the protocol invites the participants to provide suggestions, opportunities for improvement, and preferences in relation to change management in MBRU.

All participants were asked to accept the invitation to participate through electronic informed consent. The focus group session was recorded; the audio recording underwent verbatim transcription by one member of the research team (R.E.). The transcript was reviewed by the same researcher while listening to the audio recording, again, to identify and edit any errors. Each participant was assigned a unique identification number (1 through 9).

The qualitative data was inductively analyzed by three researchers (R.E., F.O., and S.D.) using a multi-staged thematic analysis framework, composed of 6 steps [33, 34]. The approach is based on constructivist epistemology and is iterative. It is widespread and is encouraged in socio-behavioral research [7, 22, 35]. The role of assuring the consistency of the analytic approach (throughout the stages of the analytic approach) was assigned to one of the three researchers (F.O.), who has thorough expertise in conducting qualitative research.

To start with, the data was studied. Then, initial codes were generated. This kept going until no new information was observed in the transcript, and hence data saturation was attained. This review led to the generation of categories of text fragments; this set the stage for the researchers to work on step three. The different ways by which these categories could relate to one another were identified through several rounds of reflections. As such, the third step culminated in the generating themes, which encapsulate groups of the previously identified categories. The fourth step involved reviewing these themes and deciding on how best they relate to one another. Then, in the fifth step, themes were labeled and assigned definitions that were of relevance to the context of the study. The sixth step constituted reporting upon the results narratively; this was done in alignment with recently published recommendations for reporting upon qualitative research [36–38].

In between the fifth and sixth step (i.e., right after the completion of the data analysis and the formation of the study’s conceptual framework, and prior to reporting upon it), the current study researchers conducted a respondent validation. The informant feedback was obtained through a 2-hour discussion conducted. During the respective meeting, the participants were shown three PowerPoint Presentation slides that included: the research questions, an explanation of the method of qualitative analysis, and the study’s conceptual framework. After showing the conceptual framework and explaining it verbally to them, the participants were given the space to reflect upon the extent of resonance between their responses to the research questions and the conceptual framework. They agreed with all the identified codes, suggesting minor changes to their definitions (within the context of the study) and how they relate to one another.

#### Quantitative

The output of this analysis guided the development of the quantitative data collection tool (a survey that relies primarily on a five-point Likert-type scale). The devised tool (S2 Appendix) evaluated the extent to which the employees agree with the perception of their leaders, along with identifying additional variables that are of relevance to the employees (with two open-ended questions: what can MBRU do to become more agile and make the organizational change processes smoother?, and what are your thoughts and reflections regarding the recent organizational changes Implemented at/ affecting MBRU?).

Based on sample size calculation, it turned out that at least 65 surveys are needed to have a confidence level of 95%. As such, 70 MBRU employees (21 faculty and 49 staff) were invited to participate by filling in an electronic version of the survey. These 70 employees were selected using Stratified Random Sampling, where the overall body of employees at MBRU (N = 186) is 30% faculty and the rest are staff. In the respective academic year (2020–2021), most of the employees (around 60%) were female and the rest were male. The employees who made the decision to fill the survey were asked to provide an electronic informed consent prior to participating in the study.

The quantitative data was analyzed using SPSS for Windows version 27.0. The descriptive analysis constituted of computing an overall score (i.e., across the 13 components- Table 1). Then, the mean and standard deviation for each of the components of the tool and the overall score were then calculated. For these variables, as well, the percentages of each of the five points of the Likert-type scale for each of the 13 components were calculated (Table 1).

**Table 1:** List of the 13 components of the five-point Likert-type scale.

Number	Five-point Likert-type Scale Component
**1**	I believe organizational change (be it internally or externally triggered) is good for MBRU
**2**	MBRU management sufficiently communicates before initiating organizational changes
**3**	The organizational changes are strategically aligned with MBRU goals
**4**	The reasons of the organizational changes at MBRU are clearly communicated
**5**	I have been told how the organizational changes will affect my department
**6**	I feel confident about delivering in alignment with the organizational changes
**7**	My manager is supportive of organizational changes
**8**	I am aware of how organizational changes are going to affect me
**9**	The organizational changes made in the University were necessary
**10**	There is an appropriate level of transparency regarding organizational changes
**11**	Sufficient efforts are put, within MBRU, to develop a common ground, among employees, before initiating organizational changes
**12**	MBRU employees have the competences necessary for effective organizational change
**13**	Overall, I am satisfied with how MBRU handles organizational change

Also, frequencies were calculated for each of the independent categorical variables (employee category, gender, & tenure). The validity tests of Cronbach’s Alpha and the Principal Component Analysis (PCA) were performed to ensure the internal consistency and check external variance, respectively, of the adapted tool. To select the appropriate inferential analyses tests, a test of normality was conducted for the overall score.

Since the data turned out to be not normally distributed, the Mann-Whitney test was used to compare the overall score between female and male employees, and the overall score between the two categories of employees (i.e., staff and faculty). Finally, Kruskal-Wallis test was used to compare the overall score between the three categories of tenures (Up to 1 year; More than 1 year, Up to 3 years; & More than 3 year).

## Results

### Qualitative

#### • Focus Group

Out of the 10 KOLs who were invited to the focus group session, 9 participated (i.e., 90%).

**Fig 2:**
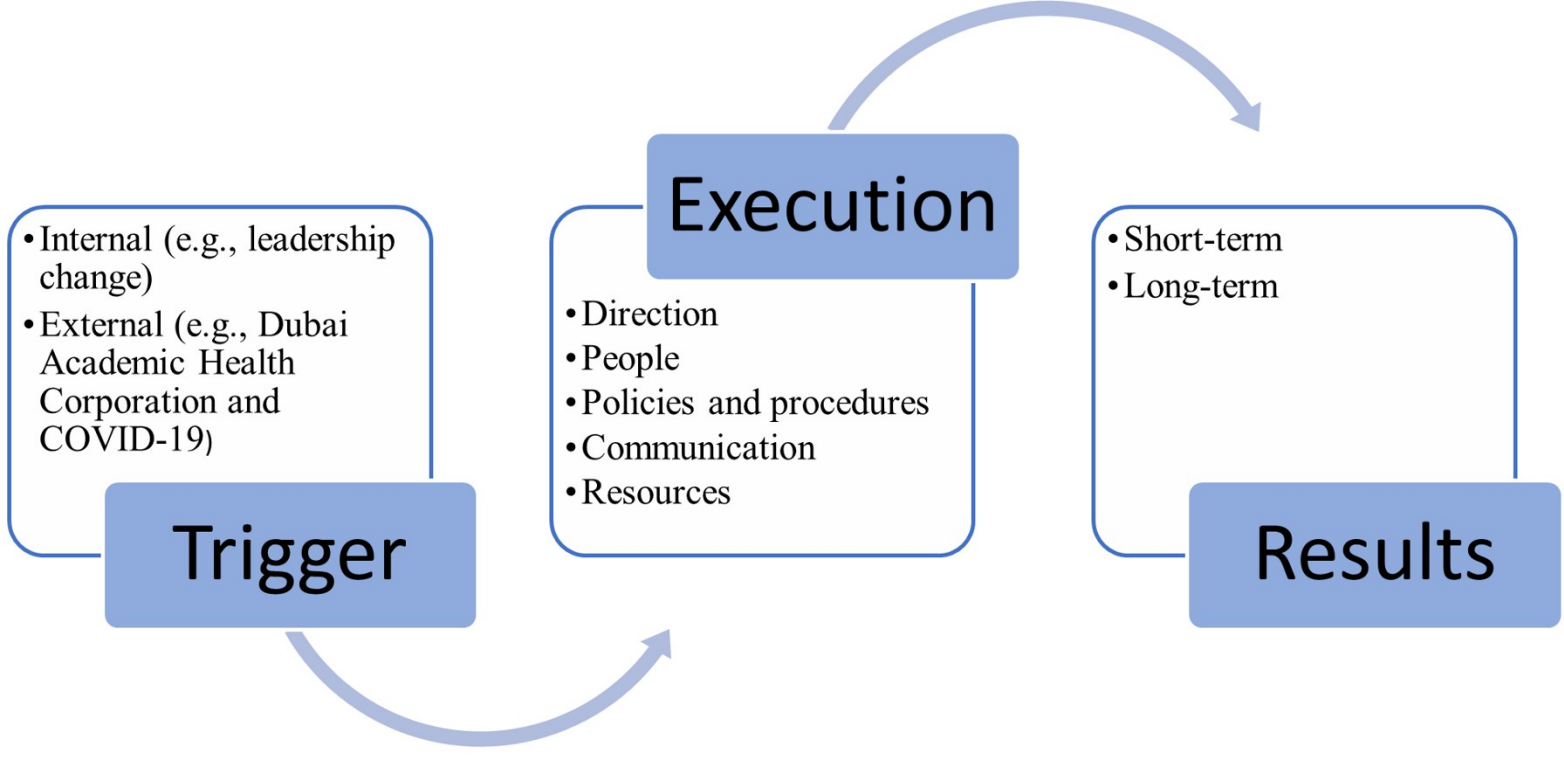
Qualitative results.

The inductive qualitative analysis of the data generated from the focus group session led to three themes, namely: Trigger, Execution, and Results, as per the study’s conceptual framework (Fig 2). Within the Trigger theme, two categories surfaced, namely: internal and external. As for the execution theme, it entailed five categories, namely: direction, people, policies and procedures, communication, and resources. The last theme was Results, and it encapsulated two categories: short-term and long-term.

#### Theme I: Trigger

The first theme included the text segments which relate to the participants’ perception of what initiated the change. They reflected upon factors that are internal to the institution.

05: “…during my tenure in this institution, I experienced a lot of changes. These changes were mainly in the governance and organizational structure, and the most recent one is infusing MBRU into the developing Dubai Academic Health Corporation. I have been relocated several times, with several changes to my reporting lines…”

06: “…there are also internal drivers to change that propel us to move forward and change. This is how universities grow. Let us say we want to offer a new program, the only way to do so is through change…"

They also mentioned situations where the change was triggered by external factors.

05: “…it is a privilege that our leaders were selected to spearhead the COVID-19 Command and Control Center. They went on a secondment, and we rose to the occasion. We genuinely missed them, their presence, and regularly engaging with them. In this case, the onset of the pandemic (which is an external stimulus) triggered a major change…”

06: “…sometimes a governing body, such as the Commission of Academic Accreditation, introduces new guidelines, which we have to adapt, and as such we change…"

#### Theme II: Execution

The second theme related to the segments of the text which highlight how the institution responded to change. This theme also encapsulated the suggestions or opportunities to improve identified by the participating employees.

03: “…building resilience is now a necessity for organizations and leaders in institutions. Resilience and agility are needed for organizations and leaders in order for change to effectively take place…”

Some employees referred to the overall direction of the institution that was set in response to the need to change. This involved several components, including strategy, leadership, and culture. Almost all the participants alluded to the importance of setting in place a strategy for the respective changes, which included pinpointing goals:

03: “…specifying the purpose and goals upfront is key to effectively handling change…”

04: “…it is important to identify the objectives of any change…”

The employees also highlighted the importance of effectively raising awareness about the direction of the change.

05: “…these points need to be direct, clear, and should be referred to repetitively… for example, our Vice Chancellor has been repeating the term ‘Academic Health System’ over the past 8 years, even prior to the establishment of MBRU. It is now part of our mindset. Afterall, ‘your vibe attracts your tribe’…”

06: “…I think it is really important to be mindful of the reasons for change: why are we changing? There also needs to be a clear-cut vision. For the change to be activated, we need to know where we want to go and how we want to get there…”

A lot of the employees reflected upon the leadership skills that are needed for the change to be effective.

04: “…the role of middle managers has been so important throughout the pandemic. They are the stakeholders who need to effectively relay/ cascade all information. So, middle management need to be fully aware of what is going on, and to be able to explain the situation and guide their employees accordingly. Afterall, the line manager is the one who ‘mingles’ the most with front-line employees…”

Within the institutional direction category, the employees also reflected upon the importance of having an agile culture.

04: “…Respect, Integrity, Giving, Connectivity, and Excellence: these are the values that we stand for…”

05: “…we need to focus on how we exhibit our values, especially during change… repetition of the message is very important; the culture is nurtured through the reinforcement of key messages…”

The participating employees reflected on how, regardless of the type of change, organizational changes affect the ways in which people go about their daily business.

They also highlighted how the success of organizational changes depends on the participation and engagement of employees. Hence, change needs to be simultaneously managed at the individual-employee and organizational levels.

02: “…agility is how quickly organizations can adapt to the necessary change; this factor is primarily reliant on the individuals and the all-encapsulating structure of the organization…”

05: “…change is enabled through acceptance and adaptation, among employees. For me, this is what helps me in moving on…”

07: “…change requires keeping an open mind. The change at the beginning may seem ‘not right’. It will also likely feel uncomfortable…”

There appeared to be a consensus that people’s buy-in is essential to effective change.

03: “…making sure everyone understands the change is important… Everybody changed quickly and understood that they need to do so swiftly. There was minimal disruption, in terms of operations and students’ learning experiences. I believe that our people handled the situation in an effective manner…”

05: “…acceptance and adaption are key for people to move on, for the change process to be expedited. We need to listen to each other. We may face disagreements and conflicts: it is important to work towards reaching a consensus. People need to reach a state of conviction prior to proceeding in the set direction… we have plenty of ‘scientific mindsets’ which generate the knowledge needed to support any decision. Supporting decisions integral to change, with evidences, smoothens and facilitates the change, and maximizes its benefits…”

The employees also thoroughly reflected on the processes that are perceived to be integral to change.

02: “…agility is exhibited when there are swift changes in processes. This requires realignment across goals, processes, and policies… Also, any change needs an operational process, which needs to be coupled with policies to ensure abiding to the respective change…”

04: “…change Management is how we deal with change. Hence, we need to learn how to approach the change…”

03: “…if you are aiming to attain a positive outcome, you need to adapt the correct processes…it is the science and art of how to handle change…”

Relevantly, the employees frequently alluded to communication, and its importance in justifying how this change will have an impact on the individuals and why it is implemented.

04: “…communication is very important, it is very important to collect and analyze evaluative data, and communicate the output of analysis…”

05: “…people need to be informed. All forms of communication, at all levels, are needed. Each personality has its own preferred way of receiving information. In a way, you need different keys to unlock different minds. We need to have variety of ways of communication to reach different personalities. Some people are ‘visual’: they need to see the message to grasp it. There are people who prefer to hear the message. So, we need to use differing types of communication in order to pass the message…”

07: “…to add to that, you know: repetition. Bite-sized messages. We had the big town hall meeting from the management. Following that with bite-sized messages reinforced the key messages in our minds. Explanation, along with repetition, is key…”

The last category within this theme related to resources that the institution needs to deploy to effectively enact change.

06: “…among the strengths of our institution was that we already had a very mature Information Technology infrastructure in place. From the academic perspective, we had a fully functioning Learning Management System, for instance. Transitioning to distance learning coupled with Working From Home was possible due to the leveraging of the existing infrastructure…”

07: “…the capacity building that took place at the onset of the pandemic constituted a major strength. We had the resources necessary to offer learning and development opportunities…”

#### Theme III: Results

The last theme related to the consequences of the change. Some employees reflected on short-term effects.

06: “…we adapted online OSCE. We had never done anything as such before. This constituted an opportunity that we leveraged during the challenging times…”

08: “…one of the challenges, integral to the transition to distance learning, was related to the student life, which really suffered in all educational institutions across the world. The students articulated this feedback in the corresponding evaluation very clearly; they missed face-to-face interactions. In terms of Working From Home, we were fine. We like the Microsoft Teams platform, we got used to it…”

Other employees highlighted long-term effects.

04: “…flexibility and adaptability were key. I had to accept. When I was told: you need to sit at home and work using Microsoft Teams. I asked myself: what is Microsoft Teams? The changes accompanying the pandemic opened our eyes to novel ways of doing things and to differing technologies. Now, if I am asked: do you prefer Working From Home? I would say: I am open to everything, working in the office or from home; it is all the same for me…”

06: “…we built resilience, on an individual and organizational levels. We were proactive about it. For example, there were a series of mindfulness sessions organized by the university counselor’s office. This will all come-in handy on the long-run…”

#### • Open-ended Questions of the Survey

To start with, a lot of the reflections shared by the employees, through the two open-ended questions of the survey, were evaluative in nature. Most of the employees praised MBRU when it comes to change management.

32-Staff: “…I consider MBRU to be agile; I trust that it effectively manages change be it induced by internal or external triggers: management had set priorities and attained targets. MBRU not only did the transition with minimum disruption to organizational processes, but also converted the challenge into an opportunity in education, research, and service provision…”

49-Faculty: “…I think the recent changes have been very positive… I feel like although we have significantly grown in number of employees, we are able to maintain the simplicity and joyfulness of MBRU’s character (which has been the case since its establishment), but with a lot more experience under our belts…”

The employees perceived change to be a requirement to the institutional advancement.

12-Staff: “…I believe that change at MBRU is for the betterment of all. I strongly believe in the upper management and the leaders at MBRU…”

37-Staff: “…change is necessary towards achieving the MBRU goals, and remaining responsive and proactive in addressing local and national needs…”

In terms of opportunities for improvement around change management, four themes were generated. The theme that surfaced the most in the survey was the importance of communication, and of improving it before and during the change.

37-Staff: “…maximize communication among staff. Continuously collect feedback from employees…”

40-Faculty: “…develop capacity to effectively communicate, and continuously reflect…”

41-Staff: “…ensure constant communication with all staff members. Keeping everyone aware of the change would help teams in better preparing and planning. With a proper plan in place, everyone will be effectively guided through the transition…”

Different ideas of how to maximize effective communication were reflected upon.

25-Staff: “…townhalls can be a good form of disseminating information about organizational change…”

28-Staff: “…evaluate the changes as they are taking place, take corrective measures when necessary, and make sure all details, in relation to the evaluation, are effectively communicated across the institution…”

The importance of communication to lessen resistance was also brought-up.

20-Staff: “…employees’ resistance can be lessened by effectively communicating all aspects of the change…”

48-Faculty: “…better communication before change will help in attaining acceptance and will increase the likelihood of positive outcomes…”

Relevant to this theme, the employees also discussed the importance of transparency.

26-Staff: “…transparency is key…”

40-Faculty: “…increase transparency, when possible…”

The second theme that surfaced, in relation to opportunities for improvement, was pertaining to preparing the employees for the change. This includes, as per the employees’ suggestions, offering learning and development opportunities that equip the employees with what it takes to effectively undergo the change.

4-Faculty: “…prepare the members of MBRU family to embrace the transformation…”

8-Staff: “…human Resources department can play a vital role to educate employees on the organizational change…”

27-Staff: “…change is inevitable, especially within MBRU. I think it is important to train the employees to accept change and make the most of the situation. Having mentors in change management would help, as well…”

The third theme that surfaced was related to maximizing employees’ engagement.

32-Staff: “…empower team members in the organization and offer them opportunities to meaningfully contribute to the change…”

33-Staff: “…get as many stakeholders as possible involved in the process to help ‘socialize’ and in turn ‘institutionalize’ the changes…”

In many instances, the participating employees attributed engagement with a supportive work environment.

35-Staff: “…encourage all employees to support each other throughout the organizational change…”

42-Staff: “…continue on fostering a motivating, engaging work culture…we are always encouraged to change to the better, be it on the personal or professional fronts…”

Within this theme, the participants reflected upon the importance of having employees play an active role in decision-making during change.

29-Faculty: “…optimize communication and engage team members in the decisions to develop shared responsibility, a sense of togetherness, and a collective commitment to the change…the organizational changes that we are experiencing are in alignment with MBRU goals…”

47-Staff: “…managers should empower their team members to take decisions…”

49-Faculty: “…involve the stakeholders from all departments in decision-making…”

The last theme, in relation to opportunities for improvement around change management, was related to optimizing the utilization of technology and automation.

21-Staff: “…automate processes to avoid any delays of work…”

36-Staff: “…to leverage technologies to facilitate the change…”

### Quantitative

Out of the 70 employees who were invited to participate, 48 responded: 36 staff and 12 faculty (Table 2).

**Table 2:** Response rate (across categories of employees) based on Stratified Random Sampling.

MBRU Employees	Total	Invited to Participate	Participants	Response percentage
**Staff**	130	49	36	73.47%
**Faculty**	56	21	12	57.14%
**Total**	186	70	48	65.71%

Among those 48 employees who responded to the electronic survey, 75% were staff and 25% were faculty, and 64.6% were female and 35.4% were male. In relation to the tenure at MBRU, 14.6% had up to 1 year of experience, 31.3% had more than 1 year and up to 3 years of experience, and 54.2% had more than 3 years of experience.

The reliability score of Cronbach’s Alpha for the tailor-made evaluation tool that captured the stakeholders’ perception was 92.8%.

The percentage of the total average was 72.3%, as per Table 3 below.

**Table 3:** Output of descriptive quantitative analysis.

Component	Mean(SD)	Percentage of the Mean	Category
**1**	4.17(0.56)	83.4%	A—SA
**2**	3.44(1.05)	68.8%	A
**3**	3.90(1.04)	78%	A
**4**	3.50(1.03)	70%	A
**5**	3.15(1.05)	63%	N—A
**6**	3.87(0.76)	77.4%	A
**7**	4.15(0.83)	83%	A–SA
**8**	3.13(0.96)	62.6%	N—A
**9**	3.75(0.70)	75%	A
**10**	3.23(0.93)	64.6%	N—A
**11**	3.21(0.92)	64.2%	N—A
**12**	3.85(0.80)	77%	A
**13**	3.69(0.88)	73.8%	A
**Overall score**	**47.02(8.62)**	**72.3%**	**A**

N = Neutral, A = Agree, and SA = Strongly Agree

According to the PCA (Kaiser-Meyer-Olkin Measure of Sampling Adequacy), 83.2% of the variance can be explained by the instrument, which means the instrument is not only reliable but also, according to Bartlett’s Test of Sphericity, valid to measure what it is intended to measure (p<0.001). Along the same lines, the Bivariate Spearman Correlations showed how changes in the components could explain the changes in the score.

**Fig 3:**
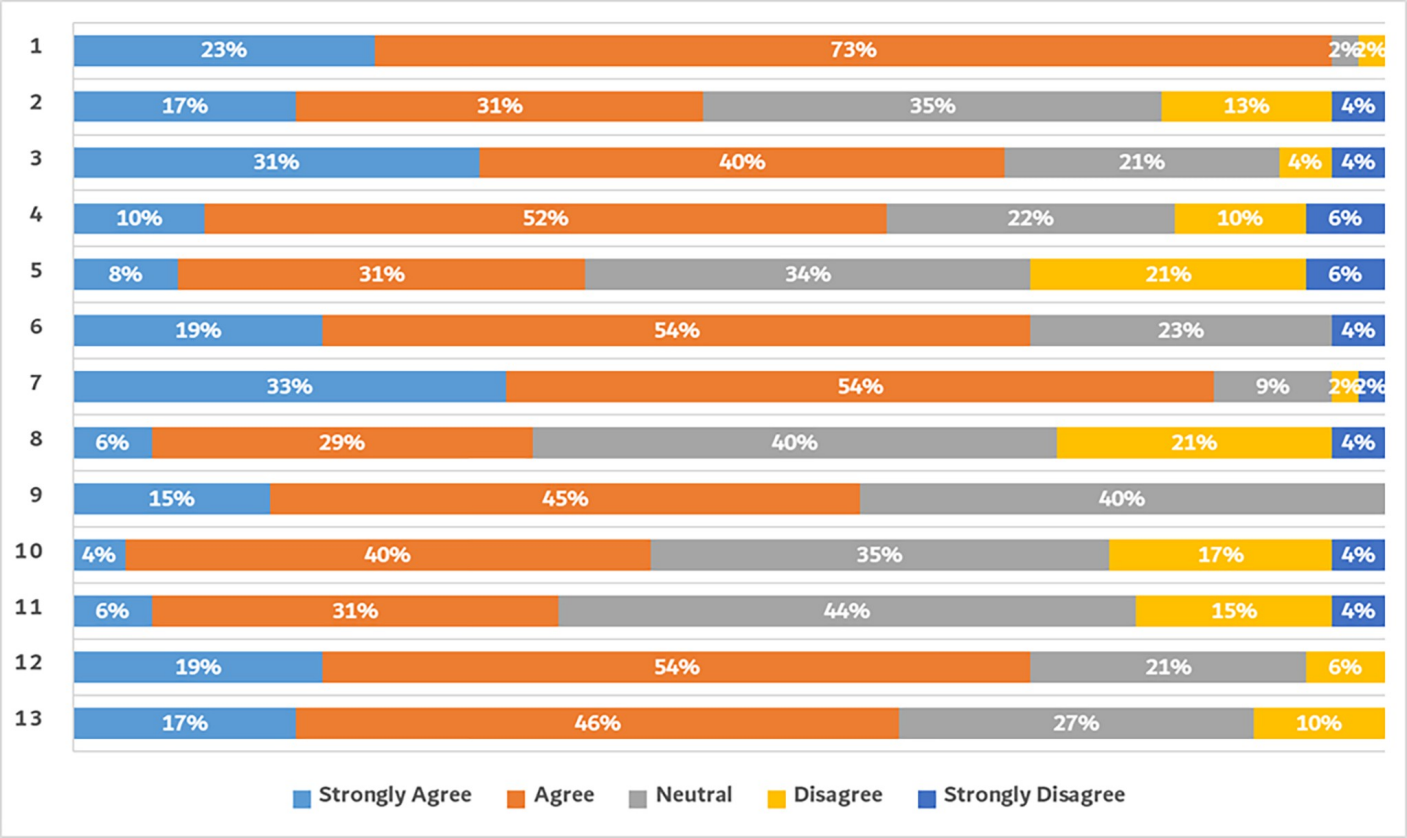
Result of questionnaire.

In terms of percentages (as per Fig 3), more than 63% of the employees agreed or strongly agreed that “Overall, I am satisfied with how MBRU handles organizational change”. Among all the components of the scale, “I believe organizational change (be it internally or externally triggered) is good for MBRU” was the most positively rated with 96% of agreement (agree or strongly agree) among employees. Followed by “My manager is supportive of organizational changes” with 87% agreement (agree or strongly agree). The component that was least rated by the participants was: “I am aware of how organizational changes are going to affect me” with only 35% agreement (agree or strongly agree) among the employees. When it came to the employees’ competences, a significant proportion of employees also agreed or strongly agreed with “I feel confident about delivering in alignment with the organizational changes” and “MBRU employees have the competences necessary for effective organizational change” (both variables with 73% agreement).

In relation to the overall score of agreement, there appeared to be no statistical significance between the differing categories of all three independent variables (staff versus faculty; female versus male; and 3 categories of tenure).

## Discussion

The current study showed that, from the perspective of higher education employees, there are three groups of variables that need to be considered when structuring an effective change management system. These groups include: Trigger, Execution, and Results. The employees reflected upon internal and external triggers to change. The external ones, especially COVID-19, appeared to have relatively influenced their workflow more. According to previous literature around the subject matter, such a change is considered to be forced upon the institution, and is contrasted with the proactive change that is usually intentional and instigated by an internal trigger [39, 40]. The employees believe that MBRU exhibited outstanding agility in responding to the pandemic. Employees’ resilience, adaptability, and openness to learn and do their day-to-day activities in different manners were evidently high. Along the same lines, previously published articles around MBRU’s rapid transition to distance learning revealed similar observations [41–43]. For example, when systematically investigating the perceptions of MBRU’s postgraduate dental learners and instructors, it was evident that they were satisfied with the respective change. It is worth highlighting that the instructors appeared to be significantly more satisfied than the learners. All in all, though, the stakeholders perceived themselves to have adapted well to the change. They were aware of the advantages and challenges of the transition to distance learning [44].

In terms of execution, it appeared that the strengths of MBRU are related to the strategy, leadership, and culture. The employees emphasized the importance of having clarity around the direction of the institution, and felt that MBRU has been doing a good job on that front. This expectation was especially relevant to internally triggered change since the employees appeared to assume that the institution and its employees have more control over it. According to Birkman [45], change requires of team members to maneuver through their day-to-day tasks despite substantial ambiguity. As such, clarity becomes of utmost importance. In this context, clarity is defined as the alignment between people and tasks to achieve team goals, along with reinforcement of cultural transparency. This alignment enables high-performing team members to march in the same direction [46]. It would be the responsibility of the leader to ensure clear definition of roles and responsibilities. Three steps to attain this clarity were specified. To start with, it is important to clearly define employee roles, and to communicate the job expectations upfront in the onboarding process. The second step is related to creating alignment, and this relates to continually clarifying roles, tasks, and decisions. Developing consensus around priorities is also crucial to maintain focus and engagement among employees, and to minimize chances of interpersonal conflict. The last step is about promoting transparency among employees, which is believed to enable an environment of accountability. This also relates to encouraging knowledge sharing. In fact, leadership is crucial to all institutions, especially universities. In academia, leadership is a central driving force in the pursuit of excellence and increased visibility. As such, effective change management starts with motivating change, followed by creating the vision, obtaining the necessary buy-in to enable the transition, and finally sustaining momentum, while moving forward [47–49].

Most of the employees praised MBRU and its leadership in relation to change management. Along the same lines, they positively rated pinpointed aspects of their first-hand experiences with change management within the institution. As such, the current study showed that, according to the participating employees, the change management within MBRU has been efficacious. Relevantly, the qualitative data showed that their leadership had clear preset targets and priorities, as a response to the onset of COVID-19, which were effectively attained. In fact, it appeared to the participating employees that the change happened with minimal disruption to the organizational day-to-day activities and processes. It is established, in the literature, that adapting to change requires leadership awareness of the internal and external environments. It is important for leaders to play the role of an environmental analyzer, learning promoter, and organizational developer. This will require for leaders to remain sensitive to environmental changes and internal barriers, and to continuously analyze these aspects of the environment, reflecting on findings, drawing implications, and establishing evidence-driven strategies to develop their organizations [50–52]. Leaders should also exhibit loyalty to their colleagues and organization, which instills a sense of security in employees, and which fosters their commitment towards the institution’s vision and direction [48, 49, 53]. A previous study recommends for leaders to deploy emotionally and morally reassuring approaches to communication with staff during the COVID-19 pandemic [54]. Paradigm shifts in education have been evident, especially from site-bounded to ‘triplisation’ (i.e., globalization, localization, and individualization), which requires leveraging of Information Technology and various networking. As such, education leaders are inevitably facing novel challenges, which is why they need to scale-up their competences to be able to effectively manage the entailed changes [55].

This study also showed that once a change is triggered, there needs to be effective communication to socialize the matter within the institution. The more engaging and collaborative the leaders are, the better it is in terms of change management [56, 57]. It was evident in the current study that employees prefer to sense their contribution to the organizational change taking place; this inspires and empowers them. Relevantly, a previously conducted study found that employees’ morale is boosted when their feedback is listened to and acted upon [53]. They also appreciate for there to be a sense of cohesion within and in between their teams, where all employees strive to support each other. There also needs to be a thorough understanding of the internal and external customers’ needs and expectations. This will enable the change to happen in alignment with the institutional values, while leveraging the institutional strengths (including but not limited to: business processes, and partnerships and affiliations) to capitalize on external opportunities [58].

In the current study, the level of satisfaction was similar between staff and faculty, female and male, and the three specified categories of employees’ tenure. The consistency of the culture at MBRU seem to be playing a buffering role where the change experiences are received and perceived similarly across employees. The concept of culture has become the foundation of quality assurance and institutional effectiveness in universities. There is ample of literature around the importance of trust, loyalty, leadership, and reputation in building quality cultures in higher education institutions [59–61]. The benefits of such cultures are not limited to improving the performance of these institutions but can stretch beyond their confines to contribute to the community-at-large and its sustainable development [62, 63].

Most of the participating employees appeared to perceive organizational change to be beneficial for the institution. The participating employees frequently alluded to the opportunities that were born amidst the challenges integral to the changes that they experienced. A lot of the employees noticed that change created the space for them to innovate, think out-of-the-box, and experiment. The benefits of leveraging existing capabilities and strengths were clear to the employees; at MBRU, they believed, the existent IT infrastructure placed the institution and its stakeholders at an advantage at the onset of the pandemic. This observation which is specific to MBRU surfaced in a previously conducted study, as well, where an inductive qualitative data analysis showed how four groups of variables, namely: People, Processes, Platform, and Policies, came together to enable the change. In other words, the interplay between the people and the processes appeared to be taking place with the existent IT platform, all of which was governed by the internal and external policies and guidelines [23, 64]. It is repetitively suggested that such transitions need to be viewed as processes, as any change in human behavior needs to take place gradually to get positive outcomes. Successes around any such transitions are attributed to intentional change management systems [65].

The employees, in the current study, specified five aspects of their experience with change management that can be improved, namely: communication and transparency, better preparing the employees for the change through capacity building initiatives, maximizing employees’ engagement and participation, and increasing the utilization of technology and automation. In parallel, it was evident from the quantitative data analysis that the level of awareness about the change, among employees, can be improved. Communication appeared to be the ‘make it or break it’ variable; the employees perceived this aspect of their experiences to be the ultimate success factors of any organizational change. The employees thoroughly reflected upon the importance of diversifying communication formats and channels, for there to be consistency and repetition, and to start early-on (ideally prior to the actual change). It was clear to the participants that change affects employees differently, and hence, they brought-up the importance of embracing employees’ individuality, as much as possible, when managing the institutional change. It is worth highlighting, over here, that surprisingly none of the participating employees referred to financial- or budget-related matters. Relevantly, a previously conducted study showed that perceived transparent communication and employee engagement constitute key mediators between perceived authentic leadership and individual employee behavioral outcomes. These findings reinforce the value of transparent organizational communication in cultivating relationships with and fostering engagement of an organization’s stakeholders [66]. Along similar lines, another study showed that employees need to be ready for change intentionally, emotionally, and cognitively [67]. Moreover, another related study proposed a model that shows how authentic leadership, transparent organizational communication, and employee engagement significantly affect employee trust. The practical implications of this study include encouraging communication managers and organizational leaders to proactively develop authentic leadership and transparent communication skills, strategies, and tactics among their employees [48]. It also suggests creating a motivating, nurturing, and transparent organizational environment which contributes to employee engagement and trust [68].

The tailor-made data collection tool introduced in this study turned out to be reliable and valid. As such, it would be recommended for other institutions, which are similar MBRU, to deploy this tool to better understand their employees’ perception of change management in their respective institutions. There are several validated tools that can be used to evaluate the perception of employees of institutional change [69–71]. None of these, however, are contextualized to a higher education institution that is undergoing several, diverse changes and validated after a worldwide crisis (i.e., the pandemic).

This study is characterized by a few limitations that are worth shedding light on. To start with, although this study offered plenty of thorough insights into organizational change management in higher education, the findings can only apply to institutions that are contextually similar to MBRU. As such, it would be recommended for follow-up studies to include a representative sample of universities across the MENA region. In addition, the survey response rate was low, which might have affected the reliability of this component of the current study. Fortunately, by nature of the mixed methods design, the findings derived from the quantitative data were effectively integrated with those of the qualitative component of the study. For upcoming studies, it would be of added value to run an investigative/ deductive, cross-sectional study, which is purely quantitative and includes several randomly selected institutions, to be able to understand the associations in between the variables would be leveraged to further reinforce decision-making. Lastly, although as shown in the study the preset focus group protocol allowed for the identification of opportunities for improvement, from the employees’ perspective, and that the consent form effectively assured the participants of anonymity and data confidentiality, there is still a chance for social desirability bias to have affected the entailed conversation. Afterall, the KLOs of any one institution to prefer to maintain a positive, professional image among each other. As such, it would be useful for exploratory researchers conducting work within higher education institutions to consider deploying one-to-one semi-structured interviews as the qualitative tool of choice as opposed to focus group sessions.

## Conclusion

In conclusion, this study highlights the importance of having agile leadership, compounded with clarity in strategy and communication, and a culture built on trust and loyalty, when it comes to organizational change. The current study specifies, from the perception of higher education employees, the triggers to change, along with key components of the organizational change execution and results. This study also introduces a valid, contextualized evaluation tool that can be leveraged by other higher education institutions to measure the perception of their employees regarding the efficacy of their change management protocols.

## Supporting information

S1 Appendix Focus group protocol. (DOCX)

S2 Appendix Survey protocol. (DOCX)
