# Supplementary material for: Change management in higher education: A sequential mixed methods study exploring employees’ perception
Source: PLoS One. 2023 Jul 21;18(7):e0289005. doi: 10.1371/journal.pone.0289005 (PMC10361480; doi:10.1371/journal.pone.0289005)
Supplement: S1 Appendix — (DOCX) [file pone.0289005.s001.docx]

**APPENDIX 1**

Change Management in Higher Education

(Virtual) Focus Group Protocol

- 10 minutes: Please share your **general understanding** about **key concepts** related to managing change in institutions.
  - Change management (from an Organizational Behavior perspective)
  - Agility (leadership, within teams)
  - Resilience
    - Organizational
    - Individual
    - Resistance to change
- 5 minutes: Change Management in MBRU
  - What constitutes change when it comes to MBRU?
    - Internal
    - External
  - How has MBRU been managing change?
- 10 minutes: Reflecting on **previous experience** with change at MBRU (since the inception of the university/ during COVID-19 times).
  - Strengths (i.e., what worked well?)
  - Weaknesses (i.e., what did not work well?)
  - Opportunities (i.e., how can we improve?)
  - Threats/ Risks (i.e., what external factors may impede the individuals or organization adaptability to change?)
- 20 minutes: Please reflect on your suggestion for effective change management at MBRU (including new techniques, means to enable employees, and preferences- leadership, middle management, and front-liners).
  - What **communication** mechanism(s) you believe is most effective during times of change?
  - How **involved** do you suggest for team members (middle management and front-liners) to be involved in decision?
  - How best to express **urgency** to obtain the right **buy-in**? (time management, People; Processes; Policies; Place/ Physical setting/ Platform
