# Supplementary material for: Change management in higher education: A sequential mixed methods study exploring employees’ perception
Source: PLoS One. 2023 Jul 21;18(7):e0289005. doi: 10.1371/journal.pone.0289005 (PMC10361480; doi:10.1371/journal.pone.0289005)
Supplement: S2 Appendix — (DOCX) [file pone.0289005.s002.docx]

**APPENDIX 2**

Change Management in Higher Education

You are invited to voluntarily participate in this research study which revolves around organizational change management at MBRU (e.g., changes in governance structure and transitioning to work-from-home and distance learning due to the onset of COVID-19). Your participation involves going through the survey questions and filling them up which is expected to take up to 10 minutes of your time.

The output of this study will be used to reinforce the decisions around change management, and maximizing employees’ engagement and agility. This will benefit MBRU and also the community- at-large. Please be assured that the obtained information will be kept confidential.

**Please make sure to read the consent form attached in the corresponding email before you proceed to filling the survey.**

* Required

# Which category of employees do you belong to? *


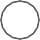

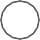
 Staff Faculty

# Gender: *


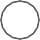

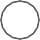
 Female Male

# Tenure at MBRU *


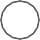
 Up to 1 year


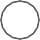

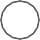
 More than 1 year, up to 3 years More than 3 year

# Please choose the appropriate answer responses for the following: *

|  | Strongly Agree | Agree | Neutral | Disagree | Strongly Disagree |
| --- | --- | --- | --- | --- | --- |
| I believe organizational change (be it internally or externally triggered) is good for MBRU |  |  |  |  |  |
| MBRU management sufficiently communicates before initiating organizational changes |  |  |  |  |  |
| The organizational changes are strategically aligned with MBRU goals |  |  |  |  |  |
| The reasons of the organizational changes at MBRU are clearly communicated |  |  |  |  |  |
| I have been told how the organizational changes will affect my department |  |  |  |  |  |
| I feel confident about delivering in alignment with the organizational changes |  |  |  |  |  |
| My manager is supportive of organizational changes |  |  |  |  |  |
| I am aware of how organizational changes are going to affect me |  |  |  |  |  |
| The organizational changes made in the University were necessary |  |  |  |  |  |
| There is an appropriate level of transparency regarding organizational changes |  |  |  |  |  |
| Sufficient efforts are put, within MBRU, to develop a common ground, among employees, before initiating organizational changes |  |  |  |  |  |
| MBRU employees have the competences necessary for effective organizational change |  |  |  |  |  |
| Overall, I am satisfied with how MBRU handles organizational change |  |  |  |  |  |

# What can MBRU do to become more agile and make the organizational change processes smoother? (Please specify two suggestions) *

1. What are your thoughts and reflections regarding the recent organizational changes Implemented at/ affecting MBRU? *
